# Robust Channel Allocation with Heterogeneous Requirements for Wireless Mesh Backbone Networks

**DOI:** 10.3390/s18082687

**Published:** 2018-08-16

**Authors:** Pangun Park, Bang Chul Jung, Hyungjoo Lee, Dae-Jin Jung

**Affiliations:** 1 Department of Radio and Information Communications Engineering, Chungnam National University, Daejeon 34134, Korea; 2 Department of Electronics Engineering, Chungnam National University, Daejeon 34134, Korea; bcjung@cnu.ac.kr; 3 Agency for Defense Development, Daejeon 34186, Korea; lhj@add.re.kr (H.L.); djjung@add.re.kr (D.-J.J.)

**Keywords:** channel allocation, partially overlapped channel, mixed-integer convex problem

## Abstract

When multiple mobile sensors and actuators share a common wireless mesh backbone network of defence systems, the channel allocation mechanism must guarantee the heterogeneous link requirements under conditions of uncertainty. In this paper, a robust channel allocation mechanism is proposed by exploiting partially overlapped channels for directional multi-channel wireless mesh networks. The approach relies on a chance-constrained optimization problem, in which the objective is to minimize the spectrum usage of the network, and the constraints are the signal-to-interference-plus-noise ratio requirements of links with uncertainty. We convert the proposed integer non-linear optimization problem into a mixed-integer convex problem by using efficient transition and approximation. The optimal channel allocation is obtained by solving the proposed optimization problem which adapts to the heterogeneous link and robustness requirements. The simulation results show that the proposed method ensures the heterogeneous link requirements under uncertain conditions while minimizing the spectrum usage of the network.

## 1. Introduction

Modern defence systems heavily rely on wireless backbone networks to collaborate and share the critical information obtained by wireless sensors and actuators, such as unmanned ground vehicles and unmanned aerial vehicles [1]. Wireless mobile sensing and actuation systems are effective infrastructure for surveillance and detection over a specific battlespace, since they provide significant benefits, such as simple deployment and maintenance, low installation costs, and high mobility [2,3,4]. The major information assets, communication, commands, and control must be reliably delivered over wireless backbone networks to support critical decisions with a radical and challenging set of defence requirements. Motivated by recent predictions of mobile sensors, unmanned ground vehicles, and unmanned aerial vehicle quantities [2,5], the channel allocation of multi-channel wireless mesh networks has become a more challenging task, since the usable radio spectrum is still a precious natural resource and the link capacity requirement has become more heterogeneous. In particular, the high bandwidth streaming video with low latency has become an essential component in the modern warfare. Furthermore, robust performance is also critical, due to unpredictable warfare, including malicious interfering and jamming attacks in deployed fields.

**Figure 1:**
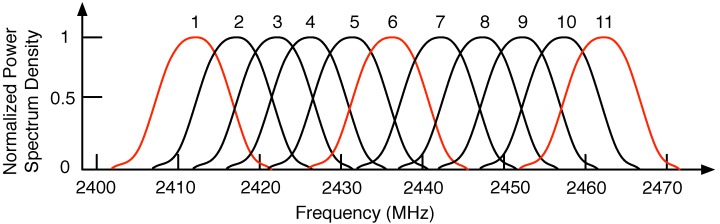
IEEE 802.11 b/g frequency spectrum diagram.

During previous years, many channel assignment algorithms have been proposed for wireless mesh networks [6]. However, most previous works defined the channel as a path of information flow which is completely isolated from other paths of wireless networks. Thus, these works only considered the non-overlapped frequency channels for the channel assignment problem. In contrast to previous works, many communication standards support the partially overlapping channels, since the energy of wireless signals is only concentrated over a narrow range of frequencies. For instance, while the IEEE 802.11 b/g standard supports 11 partially overlapping channels for transmission, the number of non-overlapping channels is only three, as shown in Figure 1. By exploiting all 11 channels in a systematic approach to avoid the interference among adjacent channels, it is possible to achieve a higher throughput than that achieved when restricting ourselves to three orthogonal channels. Recent studies show the potential for the partially overlapping channel to improve the spectrum efficiency with respect to one only using non-overlapped channels [7]. However, it is essential to carefully plan this, since adjacent channel interference may severely degrade the network’s performance.

The focus of this paper is to propose a robust channel allocation mechanism by exploiting partially overlapped channels to optimize spectrum utilization while meeting the heterogeneous link requirements for directional multi-channel wireless mesh networks. We first describe an interference model of partially overlapping channels, and then present the optimization problem of how such a model is effectively applied to the channel assignment problem. Finally, we evaluate how such optimization approaches improve the spectrum efficiency and the heterogeneous link requirements.

## 2. Related Works

Since partially overlapped channels can lead to better utilization of the spectrum and throughput improvement [7,8], there has been a growing interest in exploiting partially overlapped channels to improve network performance. Most works have focused on exploiting the assignment of partially overlapped channels to reduce interference.

In ref. [7], the authors used a binary indicator value to model the presence or absence of interference in the wireless environment. The binary indicator function was applied to evaluate the conflicts between links when different partially overlapped channels were used. The function relies on the physical distance between the nodes belonging to links, with reductions being due to the interference cost function. Even though the proposed algorithm is based on a discrete optimization problem, the solutions may not be optimal due to its heuristics.

A simple greedy algorithm was proposed to exploit the partially overlapped channel assignment problem by considering dynamic traffic in refs. [9,10]. Since the traditional conflict graph does not properly model the interference among partially overlapped channels, a modified weighted conflict graph is proposed. The edge weight in the weighted conflict graph represents the minimum channel separation that two links must have so that they will not interfere with each other. A partially overlapped interference graph is used to model interference between links in ref. [11] which is essentially the same as a weighted conflict graph. The objective of the formulated channel assignment problem is to minimize the total number of interfering link pairs or to minimize the maximum link interference.

In ref. [12], Cui et al. proposed an interference model to separate the adjacent channel considering the distance between the two access points in order to minimize the total interference while maintaining the network connectivity. A similar approach was applied to a traffic-irrelevant channel assignment algorithm to minimize total network interference [13]. The interference range of two links with channel separation was used to compute the binary conflict model for wireless mesh networks. In ref. [14], the IEEE 802.11 management framework was proposed to reduce the energy consumption and the mutual interference of densely deployed access points. In order to balance the energy consumption and the network capacity, a backtracking Tabu search algorithm was applied to determine the active access point selection and partially overlapped channel allocation. In ref. [15], a game theoretical approach was used to develop the distributed channel assignment algorithm for partially overlapped channels. They derived the upper bound for the price of anarchy of the proposed approach for multi-radio and multi-channel networks. Furthermore, there has also been research into partially overlapped channel assignment for scenarios in the absence of information exchange. A graphical game and uncoupled learning-based distributed partially overlapped channel selection was proposed in ref. [16].

Most channel assignment algorithms [7,8,9,10,11,12,13,14,15,16] use the conflict graph to consider the partially overlapped channel separation and distance of paired links. However, this approach has a fundamental limit to capture the performance of the signal-to-interference-plus-noise ratio (SINR) link due to the approximation of the cumulative terms of other interferences. The underlying conflict graph is critical for the overall performance of these algorithms, in practice. In contrast, our channel assignment approach explicitly relies on the SINR link model without any conflict graph approximations. By considering the SINR model, we formulate the optimization problem to efficiently handle the heterogeneous link and robustness requirements. To the best of our knowledge, this paper is the first study to formulate a chance-constrained optimization problem to minimize the spectrum usage while guaranteeing the link SINR requirements under uncertain conditions.

## 3. System Model

We considered a multi-channel, multi-hop wireless mesh network consisting of a number of stationary wireless mesh routers. We present the network as a directed graph (G(N,E)) in which N is the set of nodes (N=|N|), and E is the set of logical links (E=|E|) in the network. Each mesh router included multiple radio transceivers to support the simultaneous communication with other routers by using different channels. The frequency band ($\left[f_{\min},f_{\max}\right]$) was divided into a set of channels with the same bandwidth (*u*). The channels were indexed from 1 to *M*, in which $M=\lfloor \left(f_{\max}-f_{\min}\right)/u\rfloor$. We assumed that a link requirement was set dependent on the traffic demands and the criticality of the network.

We applied directional antennas to restrict the interference between mesh routers at the physical layer. An ideal sector antenna pattern was applied to model the directional beamforming [17], where two different constants were set for the antenna gains of both the main lobe and the side lobe. This simple model captures the interactions between the antenna gain, the transmission range, and the half-power beamwidth. For a given beamwidth (θ), the antenna gain ($g_{\mathrm{dir}}$) is

(1) $$\begin{array}{c} g_{\mathrm{dir}}\left(\theta \right)=\left\{\begin{array}{cc} g_{1}=\frac{2\pi -\left(2\pi -\theta \right)g_{2}}{\theta } & \;\mathrm{if}\;|\theta |\leq \frac{\omega }{2}\;\left(\mathrm{main}\;\mathrm{lobe}\right)\\ g_{2} & \;\mathrm{else}.\;(\mathrm{side}\;\mathrm{lobe}) \end{array}\right. \end{array}$$

in which typically $0\leq g_{2}\ll 1<g_{1}$.

$g_{ij}^{t}$ represents the transmitting antenna gain that the transmitter of link *i* contributes to the link between the transmitter of link *i* and the receiver of link *j*, and $g_{ij}^{r}$ represents the receiving antenna gain that the receiver of link *j* adds to the link between the transmitter of link *i* and the receiver of link *j*. These gains are characterized by Equation (1). When a node of link *i* transmits with a given power ($p_{i}$), the power received by the receiver of link *j* from the transmitter of link *i* is $p_{i}g_{ij}^{t}g_{ij}^{c}g_{ij}^{r}$, in which $g_{ij}^{c}$ is the channel gain. We used a distance-based pathloss model with Rayleigh fading as the channel gain. Thus, the link gain could be asymmetric.

**Figure 2:**
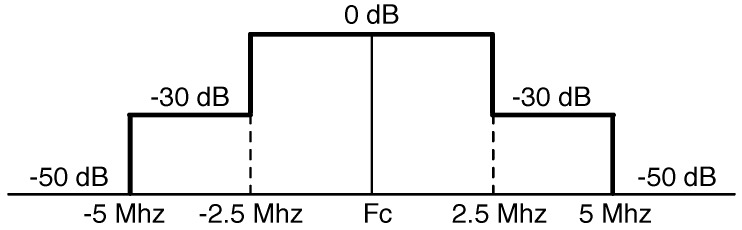
Transmit spectrum mask.

Figure 2 illustrates the transmit spectrum mask of our experimental transceiver. Many communication standards, such as 802.11 [7] and 802.15.4 [18], have a similar spectrum mask shape. The guard band that separates the two center frequencies is 62.5 KHz. This obviously leads to possible overlapping the adjacent channels and causes data collisions if it is not carefully planned.

When the transmitter and the receiver use different channels, the received signal is attenuated by the interference factor (I-factor) due to the effect of the partially overlapped channels [7]. Let us denote the power spectral density function of the transmit signal by $S_{t}\left(f\right)$, and the frequency response of the band pass filter of the receiver by $B_{r}\left(f\right)$. The I-factor between the transmitting frequency ($f_{t}$) and the receiving frequency ($f_{r}$) is defined as

(2) $$\begin{array}{c} \int_{-\infty }^{+\infty }S_{t}\left(f\right)B_{r}\left(f-\tau \right)\;df \end{array}$$

in which $\tau =f_{t}-f_{r}$. Thus, it is the cross-correlation of two functions of $S_{t}\left(f\right)$ and $B_{r}\left(f\right)$ separated by τ. As |τ| increases, the I-factor decreases due to the reduction of the overlap.

**Figure 3:**
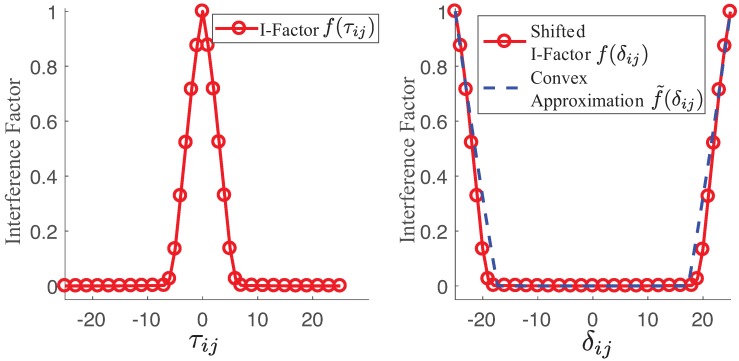
Interference factor (**left**) and its converted model as the convex function (**right**).

Let us consider two channels $x_{i}$ and $x_{j}$ in which i,j∈E and $x_{i},x_{j}\in \left\{1,\ldots ,M\right\}$. The discrete I-factor $f(\tau_{ij})$ is computed by Equation (2) by replacing $S_{t}\left(f\right)$ and $B_{r}\left(f\right)$ with channels $x_{i}$ and $x_{j}$, respectively, in which $\tau_{ij}=x_{i}-x_{j}$. Furthermore, it is possible to obtain the discrete I-factor through empirical measurements [7]. Figure 3 shows our experimental measurements of the I-factor using our transceiver, similar to the one of IEEE 802.11. Note that the received power is reduced by the I-factor $f(\tau_{ij})$ [7]. If both links *i* and *j* are operating on the same channel ($x_{i}=x_{j}$), then $\tau_{ij}=0$ and $f(\tau_{ij})=1$.

We defined a successful transmission of link *i* as a signal-to-interference-plus-noise ratio (SINR) $\gamma_{i}$ above a given SINR threshold ${\underline{\gamma }}_{i}>0$. Considering the antenna gain, channel gain, and transmit power, the SINR of link *i* with the I-factor is

(3) $$\begin{array}{c} \gamma_{i}=\frac{g_{ii}^{t}g_{ii}^{c}g_{ii}^{r}p_{i}}{\sum_{j\neq i}g_{ij}^{t}g_{ij}^{c}g_{ij}^{r}p_{j}f\left(\tau_{ij}\right)+n}\geq {\underline{\gamma }}_{i}\;,\;i\in E \end{array}$$

in which n≥0 is the power of background noise. Recall that the partially overlapped channel reduces the signal strength by $f(\tau_{ij})$. Thus, it is possible to achieve good spatial re-use by using a set of partially overlapped channels.

The constraint of Equation (3) is rewritten as

(4) $$\begin{array}{c} \sum_{j\neq i}a_{ij}f\left(\tau_{ij}\right)-\frac{a_{ii}}{{\underline{\gamma }}_{i}}+n\leq 0\;,\;i\in E \end{array}$$

in which $a_{ij}=g_{ij}^{t}g_{ij}^{c}g_{ij}^{r}p_{j}$ is a constant dependent on the large-scale pathloss of the channel gain, and transmitting and receiving antenna gains.

## 4. Channel Allocation Optimization Problem

Based on the modeling of the SINR, we formulated a chance-constrained optimization problem that ensures that the probability of meeting a certain SINR constraint is above a certain level. Note that the chance-constrained approach is one of the major methods used to solve optimization problems under conditions of uncertainty. In Equation (3), the extra random variable of the denominator of the SINR model is added to capture the uncertainty.

Our objective was to minimize the spectrum usage of the network, denoted by $\bar{x}-\underline{x}$ in which $\bar{x}$ and $\underline{x}$ are the maximum and the minimum operating channels of the overall network. The chance-constrained optimization problem is

(5) $$\begin{array}{cc} \min_{x} & \bar{x}-\underline{x} \end{array}$$

(6) $$\begin{array}{cc} s.t. & \mathrm{Pr}\left[\sum_{j\neq i}a_{ij}f\left(\tau_{ij}\right)-\frac{a_{ii}}{{\underline{\gamma }}_{i}}+n\leq \epsilon \right]\geq \eta_{i}\;,\;\forall i\in E \end{array}$$

(7) $$\underline{x}\leq x_{i}\leq \bar{x}\;,\;\forall i\in E$$

(8) $$1\leq x_{i}\leq M\;,\;\forall i\in E$$

in which a vector of the decision variables (x) is the channel index of links. Equation (6) is the extended chance constraint with the SINR requirements of Equation (4) under a normally distributed random variable $\epsilon ∼N(\mu ,\sigma^{2})$. The general confidence level $\eta_{i}$ is set to 0.9,…,0.99. Each link is required to meet the minimum SINR requirement ($\underline{\gamma }$) under a normally distributed random variable (ϵ) with a given probability (η). Note that ${\underline{\gamma }}_{i}$ is dependent on the traffic demand, and $\eta_{i}$ is the robustness requirement. Equation (7) provides the minimum and the maximum channel boundaries of the operating frequencies. Equation (8) is the feasible set of channels that are dependent on the available spectrum ($\left[f_{\min},f_{\max}\right]$) and bandwidth (*u*).

The proposed optimization problems (4) are integer non-linear problems (INP) since their decision variables are integers and they include the non-linear constraint (6). Equation (6) is the non-convex constraint, since the non-convex function of I-factor $f(\tau_{ij})$ must be smaller than a certain threshold. The reformulation of the non-convex constraint of the I-factor is essential to solve the proposed optimization problem.

### Reformation of Partially Overlapped Channel Constraint

In this subsection, we first convert the non-convex constraints given in Equation (6) into the convex constraint. Then, we reformulate the integer non-convex problem (4) into a mixed-integer convex problem (MICP).

Figure 3 illustrates the fundamental idea of model conversion. Our basic idea was to horizontally translate the I-factor model. We shifted the right part of the I-factor to the left part of the *X*-axis, and vice versa. The modified horizontal axis ($\delta_{ij}$) is

(9) $$\delta_{ij}=\left\{\begin{array}{cc} \tau_{ij}-M & \mathrm{if}\;\tau_{ij}\geq 0\\ \tau_{ij}+M & \mathrm{else}. \end{array}\right.$$

Equation (9) is easily reformulated into the mixed-integer linear constraints [19]. In Figure 3, we observe that the horizontal translation of I-factor produces a shape similar to a typical convex function.

Now, we propose a convex function to approximate the shifted I-factor. In Figure 3, one of the key observations is that the I-factor is almost exponentially decreasing as $\tau_{ij}$ increases due to the decreased overlap between the power spectrum density function of $S_{t}\left(f\right)$ and $B_{r}\left(f\right)$. One side of the I-factor is efficiently approximated with a simple polynomial function ($\alpha |\delta_{ij}{|}^{\beta }$) in which α>0,β≥1.

However, the I-factor model may have a long tail if it has a large number of partially overlapped channels dependent on the specifications. The simple polynomial function may not fit the actual I-factor with the long tail well. Since we were mainly interested in the high interfering region of the I-factor ($f(\delta_{ij})\geq 0.01$) of both sides of the boundary of Figure 3, a negative constant value was added to the simple polynomial function $\alpha |\delta_{ij}{|}^{\beta }+\phi$ in which ϕ<0. In this way, we were able to fit both sides of the boundary of the I-factor ($f(\delta_{ij})$) well. Furthermore, we added the minimum positive value (ν) due to the possibility of having a negative I-factor, $\alpha |\delta_{ij}{|}^{\beta }+\phi <0$. Thus, our approximated convex model of I-factor is

(10) $$\tilde{f}\left(\delta_{ij}\right)=\max\left(\alpha {\left|\delta_{ij}\right|}^{\beta }+\phi ,\nu \right)\;,$$

in which α>0,β≥1,ϕ<0,ν>0.

Even though it is not trivial to find the model parameters of Equation (10) due to the non-linearity of the model, it is possible to optimize its parameters by using non-linear optimization techniques, since the number of parameters is only 3. Figure 3 shows that our analytical model matches the experimental results quite well.

When the convex approximated I-factor model was applied on both sides, as shown in Figure 3, it was possible to convert the non-convex constraints of Equation (6) into convex constraints. Based on the convex approximation of the I-factor given in Equation (10), we transformed the non-convex chance constraints of Equation (6) into the convex constraints:

(11) $$\sum_{j\neq i}a_{ij}\max\left(\alpha {\left|\delta_{ij}\right|}^{\beta }+\phi ,\nu \right)-\frac{a_{ii}}{{\underline{\gamma }}_{i}}+n\leq \Phi \left(1-\eta_{i}\right)\;,\;\forall i\in E$$

in which Φ() is the normal distribution function with $N(\mu ,\sigma^{2})$. Note that the expression $\Phi (1-\eta_{i})$ is a constant due to the chance constraint with $\epsilon ∼N(\mu ,\sigma^{2})$.

The solutions to INP and MICP were obtained by solving the optimization problem (4) with the SINR constraint (6) and the one replaced by Equations (9) and (11), respectively. Since the ideal optimization problem is INP, which is difficult to solve for the global optimum, the genetic algorithm was used to obtain the solution. By appling well-known techniques of mixed-integer linear programs, there have been significant improvements to the solving of MICPs [19]. We used the CVX algorithm [20] to solve the MICP. Then, the solutions of the proposed MICP were rounded to the nearest integer value corresponding to the channel index.

## 5. Numerical Results

In this section, we first evaluate the solutions of MICP with the ideal ones. Secondly, we discuss the performance improvement of the partially overlapped channels against the one with non-overlapped channels. Finally, we evaluate the performances of the solutions of both INP and MICP.

**Figure 4:**
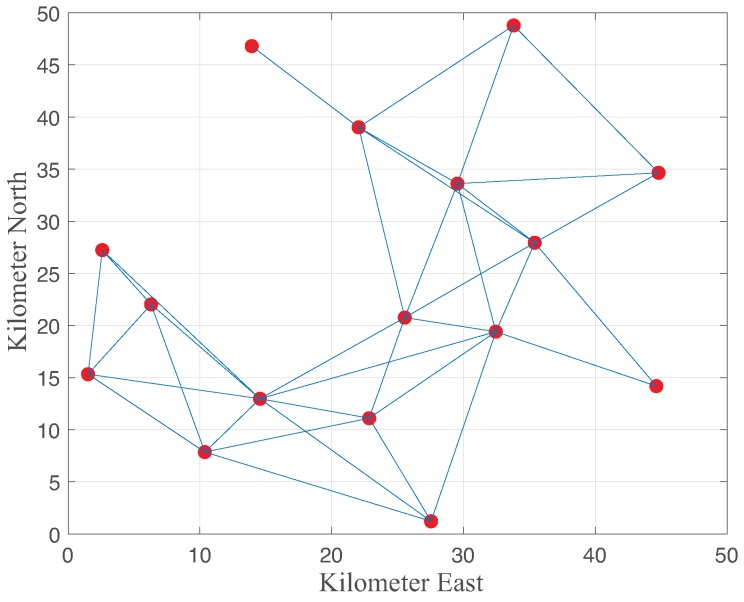
Network topology of our simulations.

We considered a static wireless network with 16 mesh routers deployed in a field of 50 km × 50 km under the maximum allowable nodal degree (8), as illustrated in Figure 4. The main simulation parameters of the paper are listed in Table 1. Note that the overall structure of the proposed robust channel allocation is applicable to the IEEE 802.11s standard for wireless mesh networks. In this network, the redundant links allow the network to operate when a node failure occurs or when a link becomes unreliable. The neighbors can quickly adapt their routing if there are failures of nodes or links. The nodes share the total available spectrum (200 MHz) with u=62.5 KHz and u=10 MHz for the partially overlapped channel and non-overlapped channels, respectively. Thus, up to M=3200 partially overlapped channels are available. Each node uses the same transmit power (1 W) with the antenna characteristics of the side lobe antenna gain ($g_{2}$) and beamwidth (θ). We considered two different topologies, namely, randomly generated topology and grid deployment with 4×4 of the mesh network. We implemented the antenna model of ref. [17] and the I-factor model of Figure 3 for the partially overlapped channels. We compared the expected values of the spectrum usage and minimum SINR of the network.

**Figure 5:**
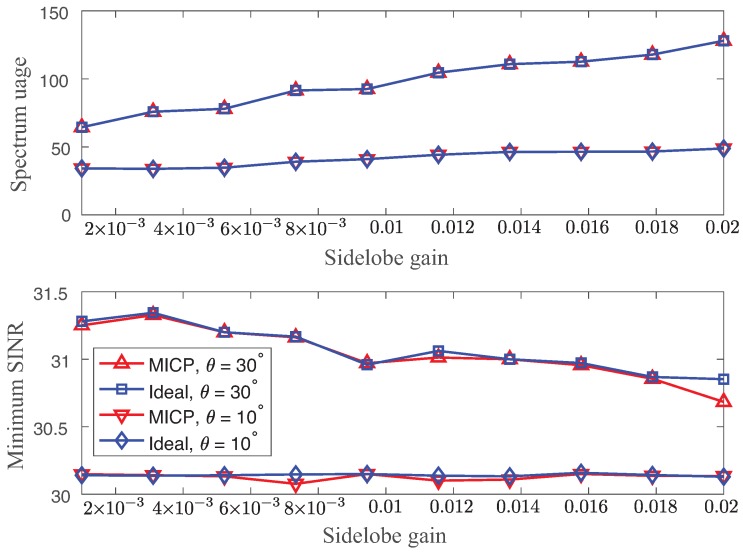
Expected values of spectrum usage (**top**) and minimum SINR (**bottom**) using the ideal solutions and heuristic solutions of mixed-integer convex problem (MICP) as a function of different sidelobe gains ($g_{2}=0.001,$ *…*, 0.02) with $\theta =10^{\circ },\;30^{\circ }$.

We first compared the ideal optimal solution and the suboptimal solution of MICP with the randomly generated topology. For the ideal solution, we assumed a continuous frequency channel selection without any constraints on the minimum bandwidth (*u*). We obtained the ideal optimal solution by using CVX. Figure 5 shows the expected values of spectrum usage and minimum SINR of the ideal solutions and the suboptimal solutions of MICP as a function of different sidelobe gains ($g_{2}=0.001,\ldots ,0.02$) with $\theta =10^{\circ },\;30^{\circ }$. We set $\underline{\gamma }=30$ dB with robustness requirements: $\sigma =10^{-8}$ and η=0.9. The suboptimal solution of MICP matched quite well the ideal optimal solution. In fact, the difference in spectrum usage was negligible. We observed some minor performance degradation of the minimum SINR of the suboptimal solution with respect to the one of the ideal optimal solution. However, it still met the minimum SINR requirement $\underline{\gamma }=30$ dB.

There are two main reasons for the good performance: the efficient approximation of the I-factor and the large number of available channels. Figure 3 shows the fairly good matching between the proposed convex approximation of the I-factor model and the real measurements. By using this model, we transformed the non-convex constraints of INP into the convex constraints of MICP. Hence, our converted MICP captured the essential I-factor of the SINR constraint without any significant loss of accuracy. Furthermore, the solutions of the proposed MICP were rounded to the nearest integer value corresponding to the channel index. This may have caused critical performance loss due to the rounding effect. However, the integer rounding effect was negligible in our cases, since we had a large number of available channels (M=3200).

In general, both solutions increased the spectrum usage while meeting the minimum SINR requirements as the sidelobe gain increased. The main reason for is that the interference increases due to the large sidelobe gain. However, the effect of the sidelobe decreases as the beamwidth becomes narrow.

**Figure 6:**
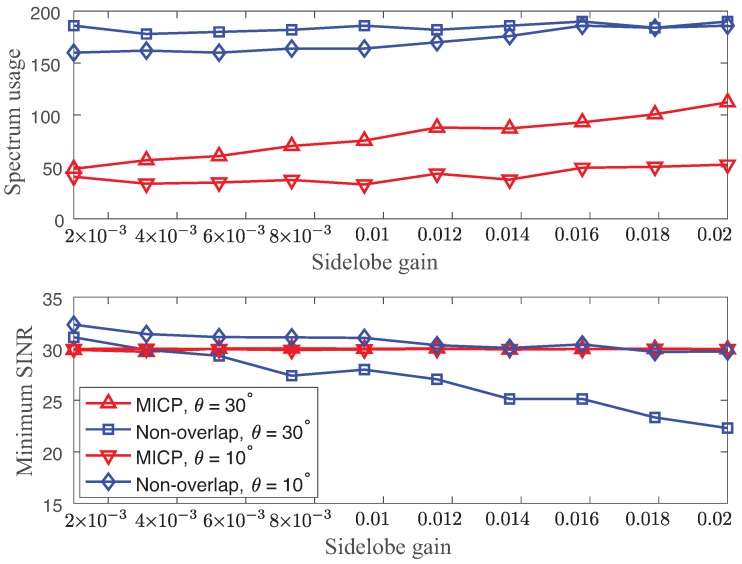
Expected values of spectrum usage (**top**) and minimum SINR (**bottom**) using the solutions of both the integer non-linear problem (INP) and MICP as functions of different sidelobe gains ($g_{2}=0.001,\ldots ,0.02$) with $\theta =10^{\circ },\;30^{\circ }$.

Secondly, we discuss the performance improvement of the spectrum efficiency and the SINR link using the partially overlapped channels against the non-overlapped channels. Figure 6 presents the expected values of the spectrum usage and minimum SINR using the MICP solutions using the partially overlapping channel (u=62.5 KHz) and the non-overlapping channel (u=10 MHz) as functions of different sidelobe gains ($g_{2}=0.001,\ldots ,0.02$) with $\theta =10^{\circ },\;30^{\circ }$. Note that the interference factor was 0.001 for u=10 MHz. We set $\underline{\gamma }=30$ dB without any robustness requirements (σ=0). The ideal solutions to the non-overlapped channels were obtained by solving the INP using an extensive search.

We clearly observed significant spectrum usage of the non-overlapped channel. In particular, the minimum SINR of the non-overlapped channel did not even meet $\underline{\gamma }=30$ dB with $\theta =30^{\circ }$ when $g_{2}>0.003$. For the narrow beamwidth ($\theta =10^{\circ }$), the minimum SINR was satisfied at the cost of large spectrum usage. Hence, the use of only non-overlapped channels led to wastage of the wireless spectrum capacity. The main reason is that the non-overlapped channels and the practical limits on the shape of transmit spectrum masks imply that there are many frequencies in which the transmitted power is lower than the maximum permissible limit, which degrades the SINR. On the other hand, the MICP solutions with the partially overlapping channel used a significantly lower spectrum while meeting $\underline{\gamma }=30$ dB over different sidelobe gains $g_{2}=0.001,\ldots ,0.02$.

**Figure 7:**
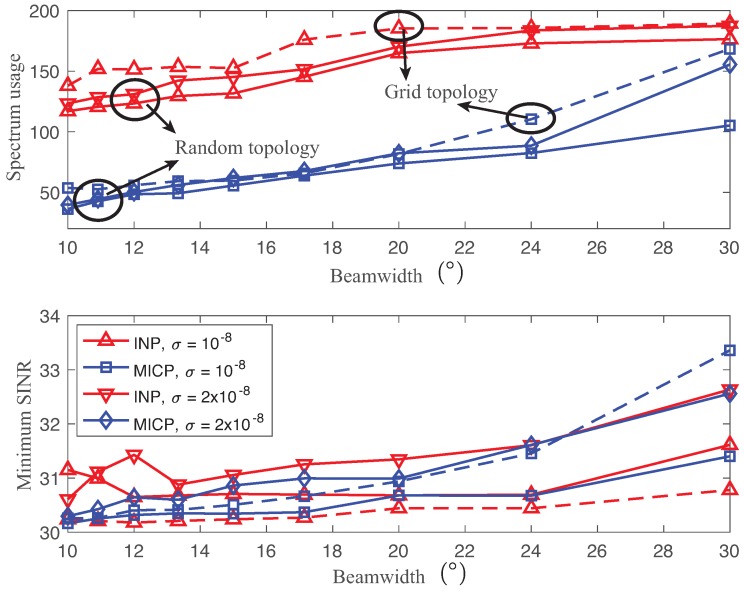
Expected values of the spectrum usage (**top**) and minimum SINR (**bottom**) using the solutions of both the INP and MICP as functions of different beamwidths $\theta =10^{\circ },\ldots ,30^{\circ }$ with $\sigma =10^{-8},2\times 10^{-8}$. The solid line and dotted line show the performance with the random and grid topologies, respectively.

Figure 7 presents the expected values of the spectrum usage and minimum SINR using the solutions of both INP and MICP as functions of different beamwidths ($\theta =10^{\circ },\ldots ,30^{\circ }$ with $\underline{\gamma }=30$ dB, $g_{2}=0.01$), and with robustness requirements ($\sigma =10^{-8},\;2\times 10^{-8},\eta =0.9$). Each link was required to meet the minimum SINR requirement ($\underline{\gamma }=30$ dB under normally distributed random variable $\epsilon ∼N(0,\sigma^{2})$) with a probability of η=0.9. A minimum SINR of links was used to show the level of confidence since the robust link performance is one of the most critical factors. The solid line and dotted line show the performance with the random and grid topologies, respectively.

In general, both INP and MICP increased the spectrum usage as the beamwidth increased due to the high interference. However, the improvement in the spectrum usage became minor for the narrow beamwidth due to the other dominant factors, such as the channel gain and antenna side lobe. Regarding robustness, MICP provided a higher minimum SINR as the uncertainty σ increased at the expense of the higher spectrum usage. Hence, it is possible to set the heterogeneous robust requirements of links based on their priorities and environment by tuning σ and η. In contrast to MICP, the minimum SINR of INP fluctuated a bit over different beamwidths due to the local minimum obtained by the genetic algorithm. Thus, we found that MICP solutions improved the spectrum usage significantly while providing a robust SINR performance using partially overlapped channels.

Overall, both INP and MICP with grid topology consumed more spectrum while providing lower SINRs compared to the methods with random topology. Since the directional links of grid topology are easily aligned due to its symmetry, it incurs more interference than the one with random topology. While the highly interfering links require the channel separation to be increased (i.e., non-overlapped channel), the channels can be closely assigned to the rest of the links for the random topology. Figure 7 also shows that MICP provides good spatial re-use of the random topology by using a set of partially overlapped channels.

**Figure 8:**
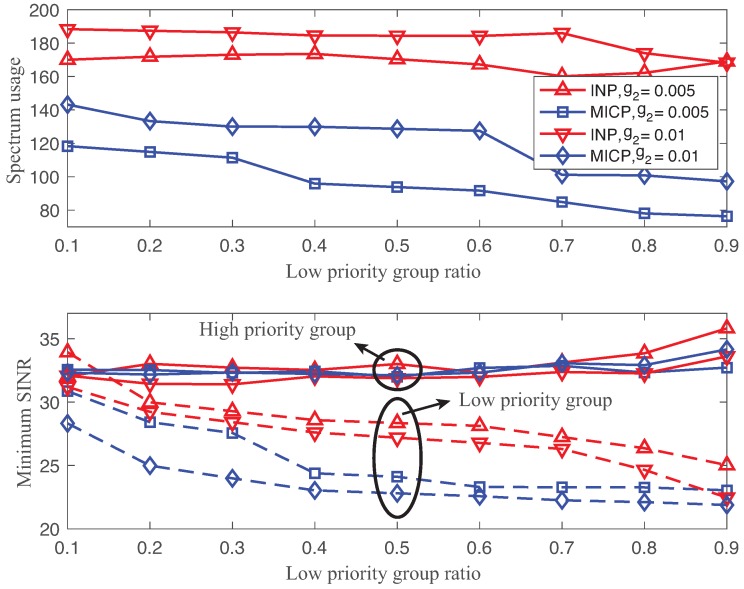
Expected values of spectrum usage (**top**) and minimum SINR (**bottom**) using the solutions of both the INP and MICP as functions of different low priority group ratios (r=0.1,…,0.9) with $\theta =30^{\circ }$, $g_{2}=0.005,\;0.01$.

One of the critical issues in channel allocation for a wireless backbone network is the heterogeneous requirement due to the radical changes of the battlespace environment. We investigated the adaptability of MICP to two different priority groups, namely, low and high priority groups, with different requirements on $\underline{\gamma }$ and η. We defined the low priority group ratio as the ratio of the number of low priority group links over the total number of links. The ratio is equal to 0 or 1 for homogeneous networks where there are only high or low priority groups, respectively. Figure 8 shows the expected values of the total spectrum usage and minimum SINR of high and low priority groups using the solutions of both INP and MICP as functions of different low priority group ratios (r=0.1,…,0.9) with $\theta =30^{\circ }$, $g_{2}=0.005,0.01$. The SINR and robust requirements of the high priority group (respective to the low priority group) are $\underline{\gamma }=30$ dB and η=0.99 (respective to $\underline{\gamma }=20$ dB and η=0.9) with $\sigma =10^{-8}$. The solid line and dotted line indicate the performances of the high and low priority groups, respectively.

We clearly observed that both INP and MICP provided a heterogeneous SINR performance dependent on the types of groups. Both solutions assigned more network spectrum to the links with the strict requirement to achieve better SINR, compared to the one with lower requirements. By comparing spectrum usage and minimum SINR, the MICP solutions performed consistently better than those of INP, since MICP significantly reduced the spectrum usage while meeting the heterogeneous requirements. The main reason for this is that MICP clearly differentiates the SINR performance based on the group requirements. We observed a greater gap in the minimum SINR between the high and low priority groups of MICP compared to those of INP. For the low priority group, MICP provided a minimum SINR performance that was slightly greater than 20 dB when r≥0.4. When r<0.4, the minimum SINR of the low priority group was greater than 25 dB. This is because the constraint of the high priority group becomes a dominant factor in the optimization problem due to the large number of links belonging to the high priority group. Thus, the simulation results clearly show that our proposed MICP guarantees the heterogeneous link requirements and efficient use of network spectrum.

On the other side, the minimum SINR of the low priority group using INP solutions was greater than 25 dB when $\underline{\gamma }=20$ dB for r≤0.8. There was an optimal channel allocation interval between links beyond which the network wasted the spectrum. Furthermore, we also observed fluctuations in spectrum usage and minimum SINR in the high priority group due to the local minimum of the INP solutions.

## 6. Conclusions

In this paper, we proposed a robust channel allocation mechanism by using partially overlapped channels efficiently for directional multi-channel wireless mesh networks. The proposed approach is based on a chance-constrained optimization problem, in which the objective is to minimize the spectrum usage of the network, and the constraints are the SINR requirements of the links with uncertainty. The optimal channel allocation was achieved by solving a mixed-integer convex problem with heterogeneous SINR and robustness requirements under uncertain conditions. The simulation results show that our proposed solution guarantees the heterogeneous link requirement and efficient use of network spectrum by efficiently utilizing the spatial re-use of the partially overlapped channels.

## Figures and Tables

**Table 1:** Main simulation parameters used in the paper.

Meaning	Value	Meaning	Value
Total spectrum	200 MHz	Guard band between two center frequencies	62.5 KHz
Number of mesh routers	16	Deployed range	50 × 50 Km
Transmit power	1 W	Power spectrum density of noise power	−184 dBm/Hz
Antenna beamwidth	10°, 30°	Sidelobe gain	0.001, …, 0.02
Maximum nodal degree	8	Minimum required signal-to-interference-plus-noise ratio (SINR)	25 dB, 30 dB
